# Avian and serpentine endogenous foamy viruses, and new insights into the macroevolutionary history of foamy viruses

**DOI:** 10.1093/ve/veaa015

**Published:** 2020-02-20

**Authors:** Pakorn Aiewsakun

*Virus Evolution*, Volume 6, Issue 1, 2020, vez057 https://doi.org/10.1093/ve/vez057

While this manuscript was in the final review stage, a related paper describing the ERV-Spuma.N-Cbo was published in the same journal (Chen *et al.* 2019).
